# Supplementary material for: A defect in myoblast fusion underlies Carey-Fineman-Ziter syndrome
Source: Nat Commun. 2017 Jul 6;8:16077. doi: 10.1038/ncomms16077 (PMC5504296; doi:10.1038/ncomms16077)
Supplement: Supplementary Information — Supplementary Figures and Tables. [file ncomms16077-s1.pdf]

SUPPLEMENTARY FIGURES

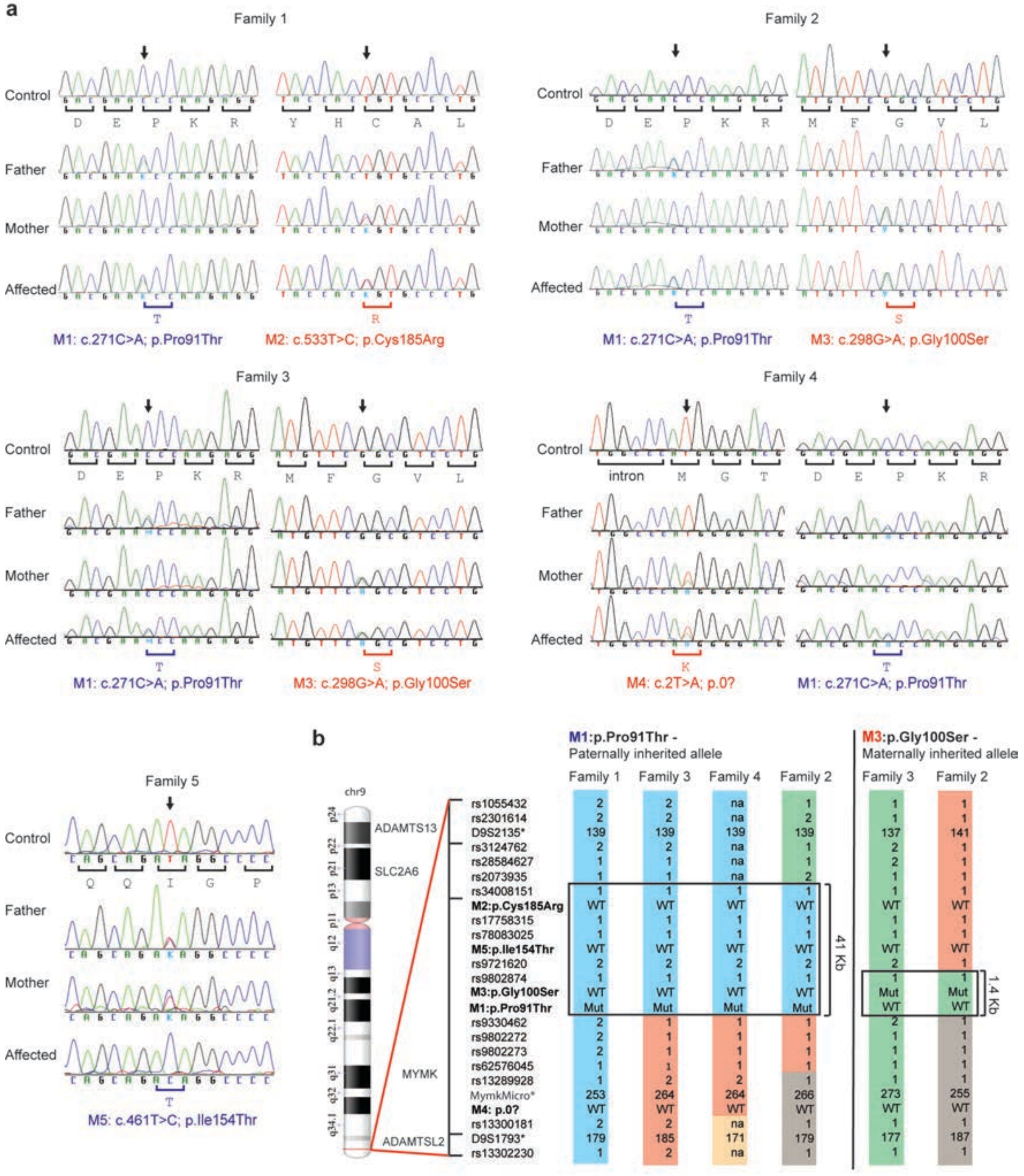

Supplementary Figure 1. Related to Figure 1. Genetic mutation analysis and haplotypes. (a) Electropherograms of relevant *MYMK* sequence in a control, the parental carriers who are each heterozygous for one variant, and an affected individual in each pedigree who harbors compound heterozygous or a homozygous variant. The altered nucleotide is indicated by an arrow over the control sequence. The corresponding WT amino acids are indicated under the control sequence, and the amino acid substitution is indicated under the affected individual's sequence. Mutations coded in blue are hypomorphic, and those in red are null. (**b**) Schematic representation of relevant haplotypes surrounding mutations M1 and M3. Left: chromosome 9 ideogram with red line indicating the position of *MYMK* at chr9qter. The bracketed region expanded to the right of the ideogram includes all of *SLC2A6* and *MYMK* and portions of *ADAMTS13* and *ADAMTSL2*. Highly heterozygous SNPs (MAF>10%) and microsatellite markers (indicated in italics followed by *) were chosen for haplotype analysis and were screened by direct sequencing. The location of each missense mutation is indicated by its designation, M1-M5. Paternal haplotypes are reconstructed for M1 in Families 1, 3, 4 and 2 (left of vertical line), and maternal haplotypes are constructed for M3 in Families 3 and 2 (right of vertical line). SNP major allele is indicated by "1" and minor allele by "2", microsatellite repeat length is provided, each mutation is indicated as WT or mutant (Mut), and 'na' denotes data not available. Common haplotypes are highlighted by the same color block, and the regions of haploidentity for M1 and M3 are boxed. Chromosome structure and gene position from NCBI based on hg19 human genome build, SNP information from dbSNP build 144. Microsatellites positions were mapped through the UCSC genome browser.

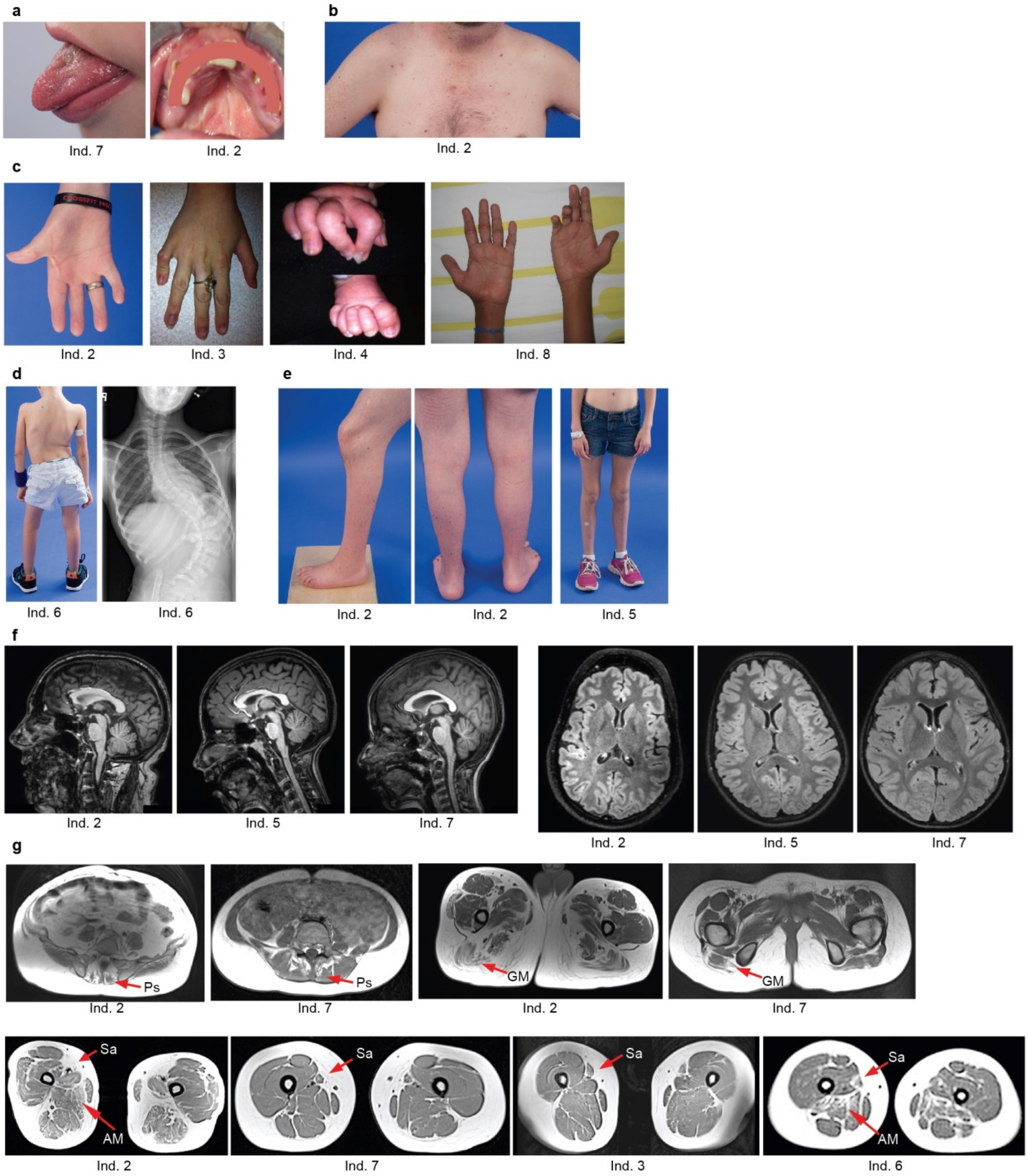

Supplementary Figure 2. Related to Figure 2. Clinical and radiological features of *MYMK*-CFZS. (**a**) Hypoglossia (Ind. 7), and high palate status post surgical repair of cleft (Ind. 2). (**b**) Pectoralis hypoplasia, right (Ind. 2). (**c**) Congenital finger (Ind. 2, 3, 4, 8) and toe (Ind. 4) contractures; hypoplastic thenar and hypothenar eminences, absent palmar and finger flexion creases (Ind. 2). (**d**) Progressive scoliosis (Ind. 6). (**e**) Generalized muscle atrophy (Ind. 2, 5, and Ind. 6 in (d)), and knee contracture with absent flexion creases (Ind. 2). (**f**) Midline sagittal 3D T1 weighted MR images (Ind. 2, 5, 7) demonstrate normal morphology of the midline structures including the corpus callosum, anterior commissure, brainstem and cerebellar vermis. (g) Axial fluid attenuated inversion recovery (FLAIR) MR images in the same individuals at the level of the thalami demonstrate normal architecture of the cortex and deep gray matter structures, normal white matter signal intensity and non-dilated ventricles. (**g**) Skeletal muscle MRI: variable involvement of the paraspinal muscles (Ps, Ind. 2, 7), gluteus maximus (GM, Ind. 2, 7), sartorius (Sa, Ind. 2, 7, 3, 6) and adductor magnus (AM, Ind. 2, 6). The most consistent finding across patients was sartorius muscle atrophy and fatty replacement. Individual (Ind.) numbers correspond to affected individuals as per Figs. 1, 2, and Table 1.

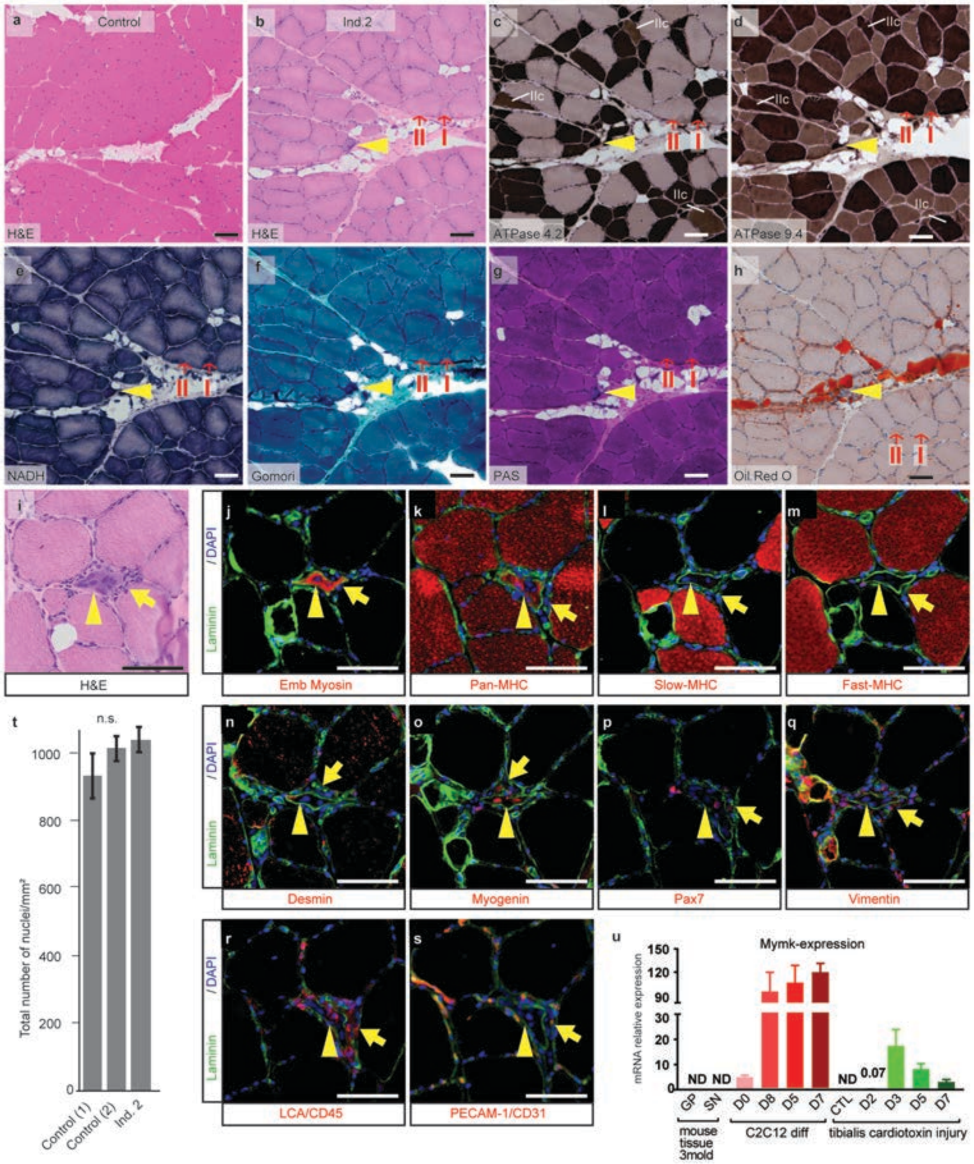

Supplementary Figure 3. Related to Figure 2. Individual 2 quadriceps muscle biopsy and *Mymk* expression are most consistent with the classification of CFZS as a congenital myopathy. (a) Age- and sex-matched control biopsy, hematoxylin and eosin (H&E) stain (14.5-year-old male, quadriceps, 10 µm thick section). **(b-h**) Clinical diagnostic panel of histochemical stains on the right vastus lateralis quadriceps muscle biopsy (12 µm thick sections) from CFZS individual 2 (male, 15 years old at time of biopsy). Within each photomicrograph, "I" and "II" point to the same type I and type II fiber, respectively, and the yellow arrowhead points to a regenerating fiber. **(b)** H&E staining shows variable fiber size with infrequent regenerating fibers (frequency of 0.14% of 3569 fibers evaluated across the biopsy) and scattered centrally-positioned nuclei. **(c)** Myofibrillar ATPase at pH 4.3 shows extensive variability in size of type I fibers with many atrophic/hypotrophic polygonal type-I fibers (dark), more uniform significant hypertrophy of type-II fibers (light), and the presence of scattered type-IIc immature fibers (intermediate staining). **(d)** Myofibrillar ATPase at pH 9.4 shows the inverse staining pattern. Regenerating fibers stain dark at both levels of pH. While there is no type II fiber grouping, there is some mild type I fiber clustering, consistent with an increased type I fiber proportion and presence of numerous atrophied/hypotrophied type-I fibers. **(e)** NADH diaphorase staining shows distributed mitochondria in the sarcoplasmic network with darker staining of type-I fibers that matches that seen in (b). **(f)** Gomori trichrome and **(g)** periodic acid-Schiff stains show no inclusions or aggregates. There are no ragged red fibers on trichrome. **(h)** Oil Red O staining shows the expected generally higher lipid content in type I fibers, consistent with a less affected muscle. **(i-s)** Magnified images of a regenerating fiber stained with **(i)** H&E, or antibodies against **(j)** embryonic myosin, **(k)** pan-myosin heavy chain (MHC), **(l)** slow-MHC, **(m)** fast-MHC, **(n)** desmin, **(o)** myogenin, **(p)** pax7, (**q**) vimentin, **(r)** LCA/CD45, and **(s)** Pecam-1/CD31 (all in red). Panels i-j are counter-stained for DAPI (blue) and anti-laminin (green). In each photomicrograph, the yellow arrowhead marks the regenerating fiber (positive for embryonic myosin, pan-MHC, desmin, and minimal vimentin, and containing some nuclei positive for myogenin, pax7, and LCA/CD45), and the yellow arrow marks cells surrounding the regenerating fiber (positive for myogenin, pax7, vimentin, LCA/CD45, and pecam-1/CD31, which marks blood vessels). There is increased endomysial tissue between fibers and laminin staining shows a "beads on a string" appearance between surrounding myofibers rather than the closely aligned parallel membrane staining characteristic of normal muscle, consistent with more closely packed nuclei and blood vessels between fibers. Note: Reported electron microscopic evaluation of this tissue described no significant increase in mitochondrial numbers or change in mitochondrial morphology. Scale bars for (a-h),(i-s)=100 µm**.** **(t)** Quantification of the total number of nuclei/unit area from Individual 2 vs. two comparable biopsies (Control 1 from (a), and Control 2, quadriceps from a 13-year-old male in green; all from 10 µm thick sections), showed no significant difference (n.s.). Statistic has been calculated using Student *t* test, mean ± SD is reported. **(u)** Expression of *Mymk* measured by qRT-PCR in adult mouse gastrocnemius muscle (GP) and sciatic nerve (SN), in differentiating C2C12 cells lines (D0-D7 pink and red bars), and in tibialis muscle before and after cardiotoxin injury (Control – D7 green bars). *Mymk* transcript expression is not detectable in mature gastrocnemius muscle, tibialis muscle, or sciatic nerve under physiological conditions. mRNA level of *Mymk* increases exponentially during C2C12 muscle cell differentiation in vitro and in tibialis muscles 3 days after cardiotoxic injury. Data reported as mean ± SEM**.**

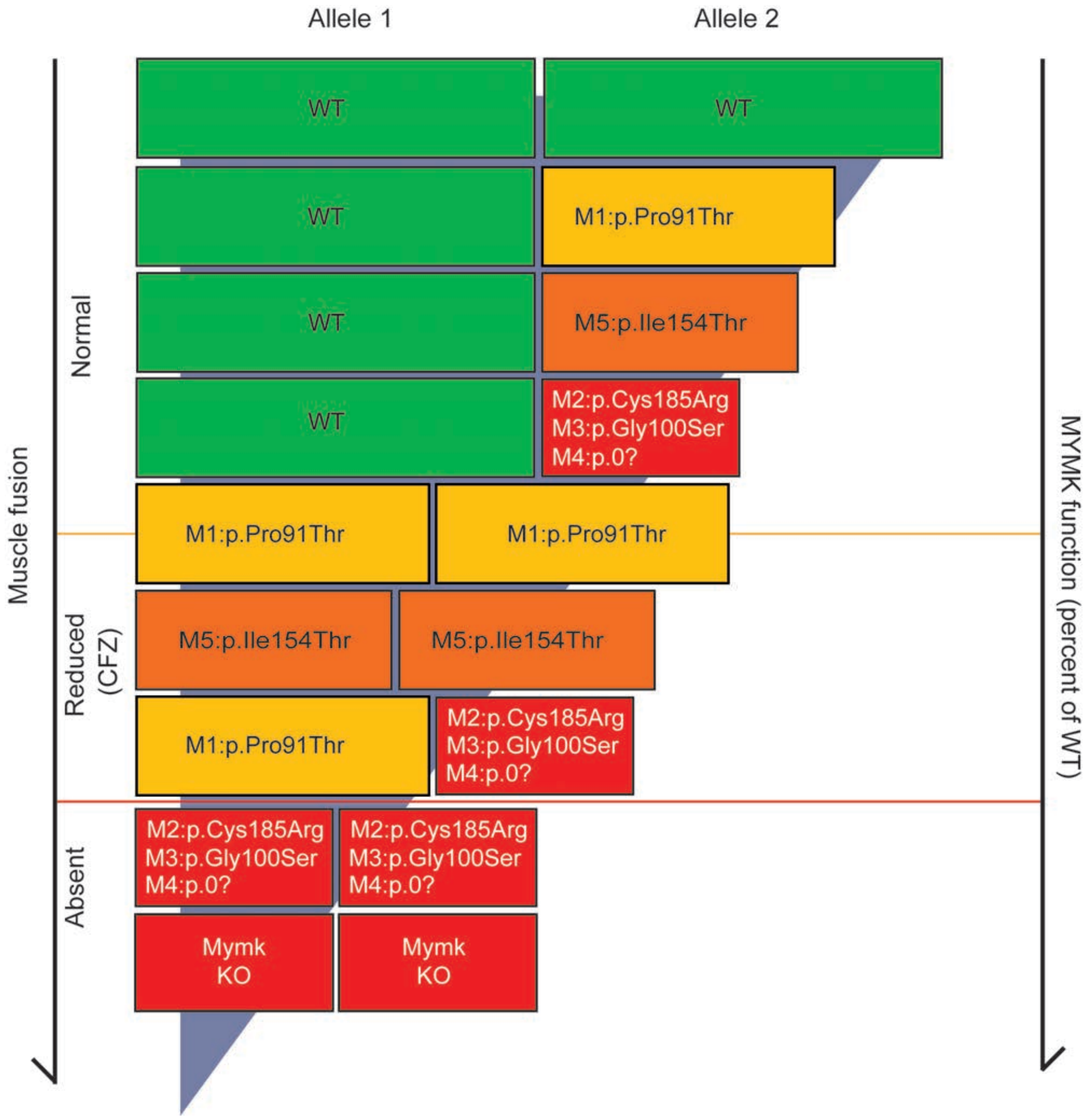

Supplementary Figure 4. Related to Figure 3. Schematic representation of the proposed genetic model for CFZS. When the degree of residual MYMK function is above a certain threshold there is normal myocyte fusion; this includes heterozygous carrier parents and potentially the individual in the ExAC database who is homozygous for the M1 allele and is presumed to have normal muscle function. When MYMK function falls below a threshold level but is still greater than 0%, there is reduced myocyte fusion and the CFZS phenotype; this includes individuals homozygous for the hypomorphic M5 allele and individuals who are compound heterozygous for the hypomorphic M1 and any one of the three null alleles (M2, M3, or M4). Absence of MYMKfunction, as observed in the *Mymk* knockout mouse model and predicted with any combination of the M2, M3, or M4 null alleles, results in absent myocyte fusion and is likely to be incompatible with life.

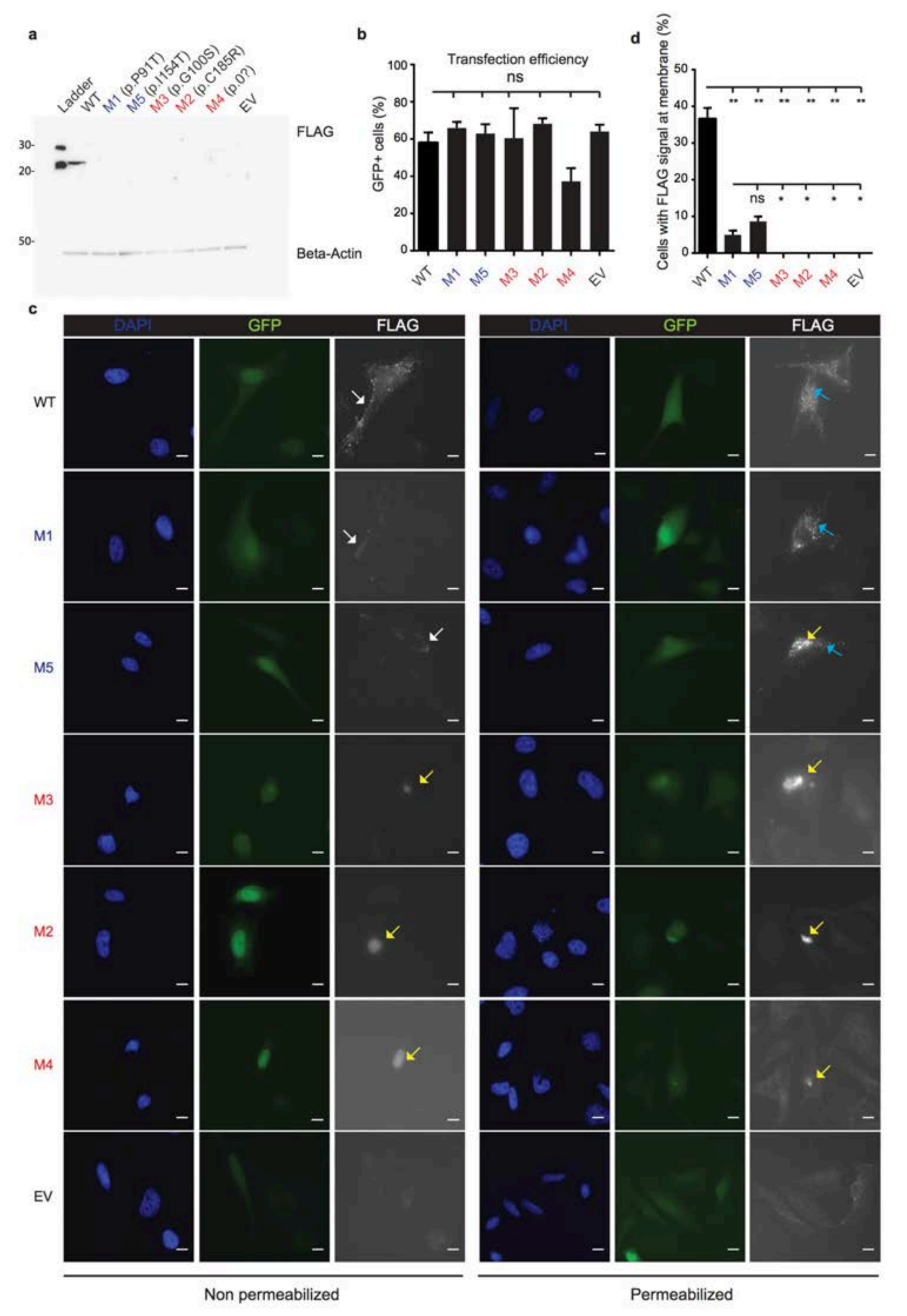

Supplementary Figure 5. Related to Figure 3. Ectopic expression of FLAG-tagged wildtype (WT) and M1-M5 mutant constructs in HeLa cells reveals reduced or absent membrane localization. (**a**) Western blot of HeLa cells transiently co-transfected with GFP and FLAG-tagged WT or mutant plasmids and revealed with anti-FLAG M2 antibody (FLAG). Expression of WT protein is observable even at short exposure times, whereas no bands are visible for M1-M5, suggesting that these alleles form an unstable protein that is rapidly degraded. Beta-actin antibody serves as a loading control. (**b**) Transfection efficiency calculated as percentage of GFP positive cells per field shows no or minimal differences in transfection level for the different plasmids. The number of GFP-positive cells was counted for at least seven random fields per condition and in three independent experiments. (**c**) Non-permeabilized (left panel) and permeabilized (right panel) HeLa cells with nuclei stained blue with DAPI, and co-transfected with GFP plasmid (green) and FLAG-tagged-WT or –mutant construct (grey). In the FLAG photomicrographs, white arrows point to membrane-localized protein, blue arrows point to punctate cytoplasmic stained protein consistent with vesicular transport, and yellow arrows point to globular cytoplasmic accumulation of protein consistent with formation of aggresomes. Row 1: FLAG-tagged WT protein localizes to the membrane in non-permeabilized cells, while punctate cytoplasmic staining is visible in permeabilized cells. Rows 2 and 3: FLAG-tagged M1 and M5 constructs localize to the membrane at a much lower frequency and with lower intensity in non-permeabilized cells compared to WT, establishing that these hypomorphic proteins can be integrated in the membrane. Punctate cytoplasmic staining is visible in permeabilized cells, as well as occasional cytoplasmic accumulation. Rows 4-6: M2-M4 have no membrane signal in non-permeabilized cells and no punctate cytoplasmic staining in permeabilized cells. Many more cells have cytoplasmic inclusions than are seen following M1 or M5 transfection. Row 7: Empty vector (EV) serves as a negative control. Scale bar, 10 µm. (**d**) Quantification of the number of cells with FLAG signal at the membrane versus total number of cells. Signal intensity is not accounted for in the quantification. At least seven random fields per condition and in three independent experiments were analyzed. Approximately 37% of WT and 5-10% of M1 and M5 transfected cells have FLAG signal at the membrane, while 0% membrane staining was observed for M3, M2, M4 and empty vector (EV). Statistics in (b) and (d): One-way ANOVA with Bonferroni correction for multiple testing. Data reported as mean ± SEM; **, $p<0.0001$; *, $p<0.05$; ns, non-significant.

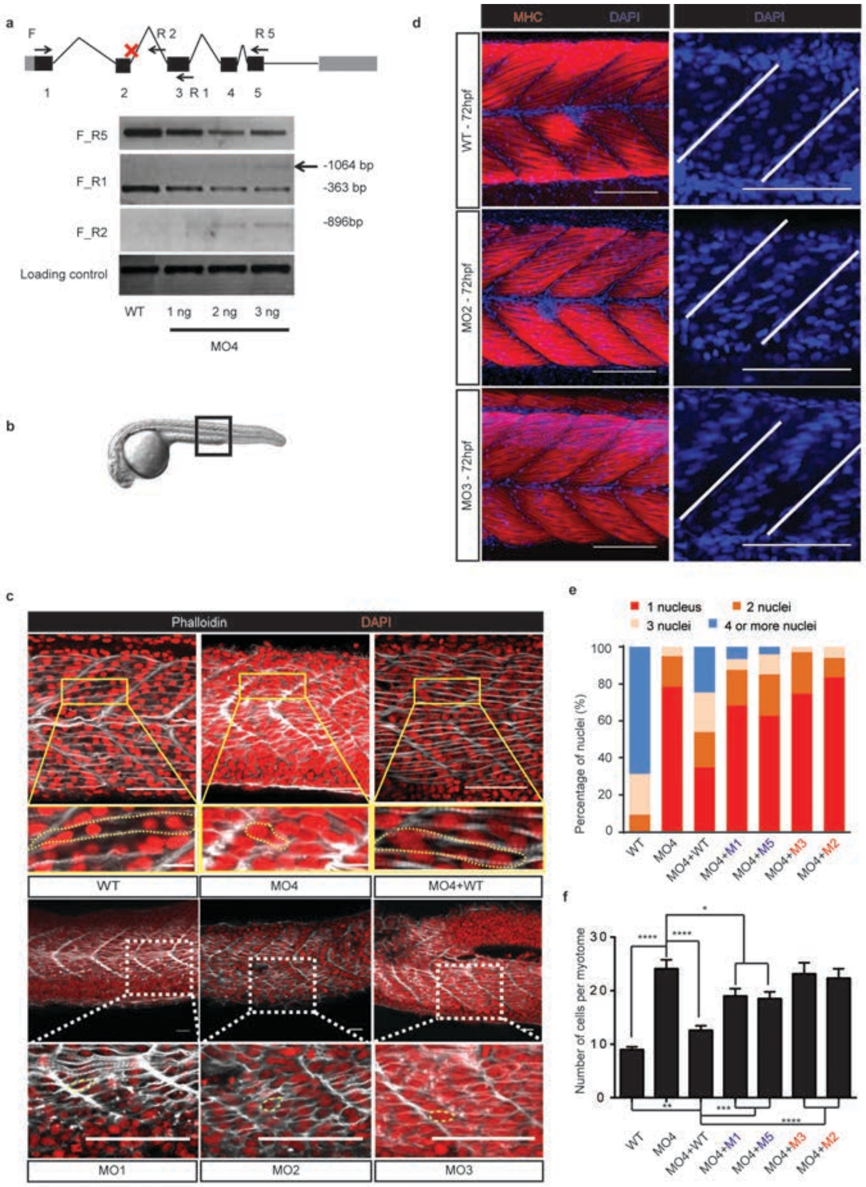

Supplementary Figure 6. Morpholino-mediated knockdown of *mymk* in zebrafish leads to incomplete myoblast fusion that is partially rescued by co-injection of WT or hypomorphic-mutant *MYMK* mRNA, but not rescued with null-mutant *MYMK* mRNA. (**a**) Splice-blocking morpholino MO4 causes aberrant retention of intron 2 in the zebrafish *mymk* transcript. Top: Schematic representation of *mymk* transcript. Grey boxes indicate the 5' and 3' UTRs, black boxes indicate exons, and connecting lines indicate introns. A red cross indicates the MO4 binding site. Arrows denote the position of forward (F) and reverse (R1, R2, and R5) primers used to amplify the WT and aberrant transcripts by RT-PCR. Bottom: RT-PCR products following amplification of *mymk* transcript with F+R5 (top), F+R1 (second row), F+R2 (third row) from cDNA of WT zebrafish and zebrafish treated with 1ng, 2ng, and 3ng of MO4. Note the appearance of the F_R2 band with increasing doses of MO4, reflecting failure to splice intron 2 out of the transcript. Note also the appearance of the 1064 bp band following amplification of F_R1 in the presence of 3ng MO4, reflecting the larger transcript. Finally, the amount of WT F-R5 and F-R1 product falls with increasing MO4, likely reflecting nonsense mediated decay. Amplification of *mafa* is provided as a control (bottom). (**b**) Brightfield picture of a zebrafish larva at 24 hpf. The black box indicates the area around somite X depicted in (d). (**c**) Lateral view at 24 hpf of control, MO1-MO4 morpholinos, and MO4 morpholino+WT mRNA injected zebrafish embryos showing the medial region of the myotome surrounding somite ten (X) as boxed in (b). Membranes of fast-twitch myoblasts are stained grey-white using fluorescently conjugated phalloidin; nuclei are stained red with DAPI. Boxed regions are shown in higher magnification below with yellow dotted lines outlining a single myofiber. WT fish have fully fused myofibers each containing up to 4 nuclei. Morpholino-treated embryos have normal appearing myotomal organization but have many single (unfused), two, and three nuclei myofibers. Co-injection of MO4 with 100 pg of human WT mRNA partially rescues the multinucleated phenotype. Scale bars: top images 50 µm, inset 100 µm. (**d**) Lateral view of AB zebrafish embryos at 72 hpf showing differentiated myofibers stained with F310 anti-fast-myosin heavy chain antibody (red) and DAPI (blue). WT embryos have well-organized myofibers aligned tightly parallel to one another and the nuclei show no clear preferential position within the myotome. By contrast, the myofibers in *mymk* morpholino-injected embryos are less well aligned, and nuclei cluster toward the center of the myotome, parallel to the myosepta (transverse white lines). Scale bars 100 µm. (**e**) Number of nuclei present in each myofiber within a single myotome adjacent to somite X in WT and MO4 morpholino-treated embryos, and in embryos co-injected with MO4 and human WT or mutant mRNA, reveal a consistent partial rescue with human WT mRNA, less rescue with the two hypomorphic alleles, and no rescue with the two null alleles. Results are normalized to total number of cells present within each myotome. (**f**) Quantification of the number of cells defined by phalloidin-stained membranes within a 2500 μm^2 area of a single myotome adjacent to somite X. *P* values were calculated by two-tailed Student's *t* test. N's for (e and f) as follows: WT n_{total}=10 (n per injection: 3/2/1/1/1/2); MO4 n_{total}=10 (1/4/5); MO4+WT n_{total} =10 (1/4/5); MO4+P91T n_{total} =9 (5/4); MO4+I154T n_{total} =8 (2/6); MO4+G100S n_{total}=6 (3/3); MO4+C185R n_{total} =6 (3/3). Within an experimental condition, each injection was on a separate day. Figure d upper panel scale bars 50 µm, inset scale bars 10 µm. Figure c lower panel scale bars 50 µm, inset scale bars 100 µm. Figure d scale bars 100 µm. See also **Supplementary Table 2**.

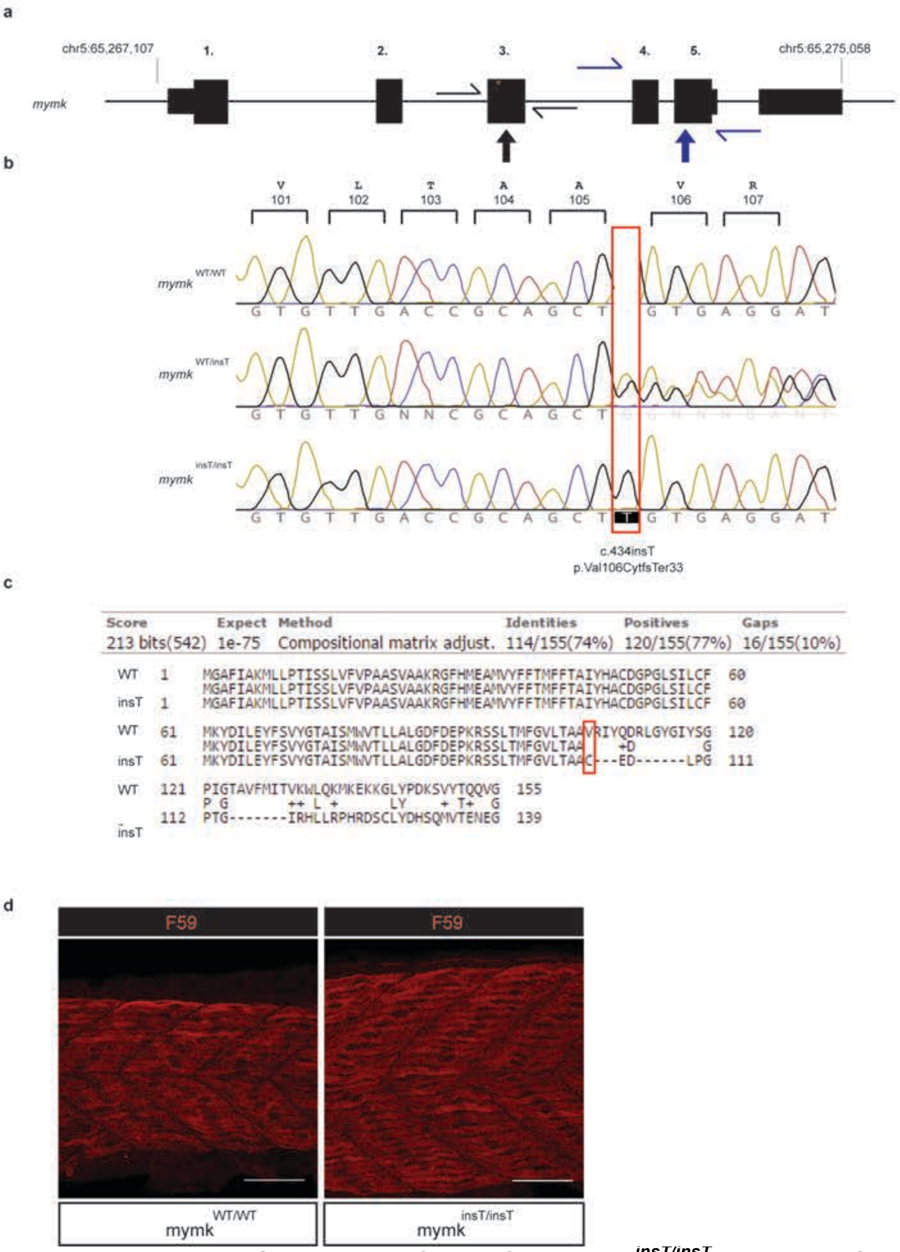

Supplementary Figure 7. Generation of zebrafish *mymk*$^{insT/insT}$ line using CRISPR-Cas9 technology. (**a**) Zebrafish *mymk* gene schematic with chromosomal position based on danRer10 genome build and exon numbering. Vertical black and red arrows indicate the target location of the sgRNAs used to generate CRISPR-KOs, and horizontal black and blue half-arrows indicate the location of the two sets of primers used to confirm the CRISPR induced NHEJ in exon 3 and 5, respectively. A red asterisk indicates the position of the frameshift, mutation at amino acid 106. (**b**) Electropherograms using primers indicated in black in (a) of WT, heterozygous and homozygous F2 *mymk*^{insT} CRISPR loss-of-function lines reveal the insertion of a second thymidine (T) after the T at position c.434. Amino acid single base letter codes and relative position of mymk WT sequence are indicated above the electropherograms. The position of the insertion is highlighted by a red rectangle. The inserted T is indicated inside the solid black box within the nucleotide sequence beneath the bottom sequence, with the altered protein sequence below. (**c**) Alignment of WT and mymk^{insT} zebrafish protein using Blast2Seq algorithm. The insertion of a T at position c.434 is predicted to substitute a cytosine (C) for a valine (V) at position 106 and generation of 33 novel amino acids before encountering a premature stop codon. (**d**) Maximum projection of Slow-twitch myofibers in mymk WT and homozygous insT at 48 hpf. Myofibers are stained for F59, staining Myosin heavy chain. Mutant appears larger because of the flattening due to less developed fast twitch-fibers. Myofibers size and orientation is similar between mutant and WT. Scale bar=50 μm.

SUPPLEMENTARY TABLES
Supplementary Table 1. Exome sequencing filtering and data

a. Exome sequencing coverage

	Family 1	Family 2	Family 3
Individuals sequenced	2 parents<br>2 affected sibs<br>1 unaffected sib	2 parents<br>2 affected sibs	2 parents<br>2 affected sibs
Total mapped reads	112,558,424 - 327,214,489	43,391,540 - 58,243,300	116,926,906- 143,334,597
Mean coverage on target	80.32 - 320.29	53.62 - 71.03	94.03-132.05
Percentage of capture target covered with >10 read depth	96.73 - 99.05	97.42 - 98.27	96.66-98.91

b1. Variant analysis and filtering of Family 1 exome data

Filter	Variant number
Total variants in proband (homozygous and heterozygous, PASS and LowQual)	1,093,451
Nonsynonymous, nonsense, frameshift, insertion/deletion, splicing variants	12,235
Global MAF ≤ 0.01 (1000 Genomes)	3,003
MAF≤ 0.01 (NHLBI ESP6500)	2,798
Present in both proband and affected sister	1,340
Recessive (homozygous and compound heterozygous) inheritance analysis (heterozygous in parents, absent or heterozygous in unaffected brother, homozygous or compound heterozygous in two affected sibs) + visual inspection of alignment on BAM files with IGV	6<br>(in 3 genes)

b2. Variant analysis and filtering of Family 2 exome data

Filter	Variant number
Total biallelic variants	126566
MAF ≤ 0.01 (NHLBI ESP6500)	77712
Global MAF ≤ 0.01 (1000 Genomes)	22552
Predicted to change encoded protein (nonsynonymous, stop, frameshift, insertion/deletion, splicing)	3397
Homozygous in both sibs; heterozygous in both parents, or compound heterozygous at a single gene in both sibs with each parent heterozygous for ≥ 1 variant in that gene	25 (in 10 genes)
Removal of probable miscalls (allele ratios > 5:1 in variants)	12 (in 7 genes)
Removal of variants with allele frequencies > 0.01 in ExAC	7 (in 5 genes)

b3. Variant analysis and filtering of Family 3 exome data

Filter	Variant number
Total Variants in the family	155878
Passing the PASS filter and QUAL filter	115065
Nonsynonymous, nonsense, frameshift, insertion/deletion, splicing variants	13153
Global MAF ≤ 0.01 (1000 Genomes)	1311
MAF ≤ 0.01 (NHLBI ESP6500)	962
MAF ≤ 0.01 (EXAC and internal database)	862
Present in both proband and affected sister	172
Recessive model of inheritance	1

c. Loci with biallelic rare SNVs shared by both affected siblings detected on exome sequencing

Family 1	Family 2	Family 3
MUC6	*FAM120B*	*MYMK*
MYMK	*OLIG1*	
ZNF366	*RHD*	
	TACC2	
	MYMK	

d *MYMK* polymorphisms identified in sequenced cohort.
* 1000 Genomes

cDNA position	Position	SNP ID	MAF (ExAC)	Zygosity
c.1-49T>C	intronic	rs13300181	0.3536	homo
c.136-144C>T	intronic	rs72779234	0.0551 (*)	het
c.136 -43T>C	intronic	rs9802273	0.2620	homo
c.136-27T>C	intronic	rs9802272	0.2523	homo
c.192C>T	p.D64D	rs777029769	0.000008	het
c.219G>A	p.G73G	rs143433318	0.0062	het
c.251-18T>C	intronic	rs11523307	0.1122	het
c.253C>T	p.L85L	rs149973300	0.001	het
c.534T>C	p.Y178Y	rs17758315	0.3914	het/homo.

e. *In silico* prediction of pathogenicity of identified variants

Code	Allele	MAF (ExAC)	Protein	PolyPhen2	SIFT	PROVEAN
M1	c.271C>A	0.0013	Pro91Thr	1.0	0.65 Tolerated	-1.12 Neutral
M2	c.553T>C	absent	Cys185Arg	0.6	0.03 Damaging	-2.29 Neutral
M3	c.298G>A	absent	Gly100Ser	1.0	0.21 Tolerated	-5.98 Deleterious
M4	c.2T>A	absent	p.0?	0.98	0 Damaging	-1.50 Neutral
M5	c.461T>C	absent	Ile154Thr	0.77	0.13 Tolerated	-2.65 Deleterious

Supplementary Table 2. Cell and nuclei counts per zebrafish myotome following *MYMK* morpholino knock-down

Sample	# fish analyzed	Average # cells per myotome	SD	Average of given number of nuclei and SD per myotome							
				1	SD	2	SD	3	SD	>3	SD
WT	10	9.00	1.55	0.00	0.00	2.07	0.87	4.93	1.04	15.30	1.81
MO3	10	24.10	4.97	18.70	4.63	3.90	1.30	1.20	1.17	0.00	0.00
MO3+WT	10	12.60	2.49	4.40	2.06	2.40	1.91	2.70	0.64	3.10	1.58
MO3+M1	9	19.00	3.82	12.78	4.42	3.56	1.71	1.11	1.20	1.22	1.03
MO3+M5	8	18.50	3.40	11.63	2.23	4.13	1.96	2.00	1.87	0.75	0.83
MO3+M3	6	23.17	4.60	17.33	4.89	5.17	1.57	0.67	0.47	0.00	0.00
MO3+M2	6	24.33	1.24	19.50	1.26	3.50	1.50	1.17	1.07	0.00	0.00

Supplementary Table 3. Primers used for PCR, sequencing and genotyping *MYMK*

Primer Name	Type	Sequence	Species	Notes
MYMK_E1_F	E1	GTGACAGGGCGAGACTCTG	H	PCR & seq
MYMK_E1_R	E1	TCCAGATAGGGGAAGGGACT	H	PCR & seq
MYMK_E2_F	E2	TGAGCGAATTCTGCTGAGGT	H	PCR & seq
MYMK_E2_R	E2	GCATCAGGGCATTTAAGAGG	H	PCR & seq
MYMK_E3_F	E3	GGCTGCCTGTCCTCTCAA	H	PCR & seq
MYMK_E3_R	E3	CGTGGAGGTGAGACATTGG	H	PCR & seq
MYMK_E4_F	E4	CCAGCAGAAGCCATCTGAG	H	PCR & seq
MYMK_E4_R	E4	ATGGTGCTGGTGGTGCTG	H	PCR & seq
MYMK_E5_F	E5	TCCTGCAGGTGTCCCAGT	H	PCR & seq
MYMK_E5_R	E5	AAGCATTTAGTGCAGCATGG	H	PCR & seq
MYMK_SNP_1_1_F	I1	AGTGCTTGCTGCATGTGTTC	H	I SNP geno
MYMK_SNP_1_1_R	I1	TAGACCTAGCCCAGCAAACC	H	I SNP geno
MYMK_SNP_1_2_F	I1	ACACGTGCACACTCAGCTTC	H	I SNP geno
MYMK_SNP_1_2_R	I1	CCACCCTCCCTGGTTTTAGT	H	I SNP geno
MYMK_SNP_2_F	I2	TGAAACCCAGACAAGGGAAG	H	I SNP geno
MYMK_SNP_2_R	I2	CAAGGAAGCACAGATGCTGA	H	I SNP geno
MYMK_SNP_3_F	I3	TCCAGCCAGTTTGAGAGGAC	H	I SNP geno
MYMK_SNP_3_R	I3	GGGTTTCAGGGTTGTGCTTA	H	I SNP geno
MYMK_SNP_4_F	I4	CCAGGATTAGTGCTGCTTCC	H	I SNP geno
MYMK_SNP_4_R	I4	AAGTGCCAAGAGAGGACCTG	H	I SNP geno
MYMK_SNP_5_F	I5	ATAGCCAGGGCACAGTGGTA	H	I SNP geno
MYMK_SNP_5_R	I5	AAAGCTGGCTGCCTACAGAC	H	I SNP geno
DS91793_F	Microsat	GGTGTGGAACCAGGACTAAC	H	Geno
DS91793_R	Microsat	CAGAGCGAGTGTAATCCG	H	Geno
MYMKMicro_F	Microsat	GGGCATGTTAGTGGAGAAAGTC	H	Geno
MYMKMicro_R	Microsat	TCTCTAAAGCTCTTGGTCCCAC	H	Geno
D9S2135_F	Microsat	AGGTCTCACTGCATACATGGG	H	Geno
D9S2135_R	Microsat	GTGGACATGTGCCTTCAGG	H	Geno

E = Exon; I = Intron; Microsat = microsatellite; Geno = genotyping
